# Examining spatiotemporal crowdsensing and caching for population-dynamic OTT content delivery

**DOI:** 10.1038/s41598-024-64589-1

**Published:** 2024-06-14

**Authors:** Hee Soo Kim, Yumi Jang, Yun Jae Choi, Hong Ki Kim, Seongcheol Kim, Sang Hyun Lee

**Affiliations:** 1 School of Electrical Engineering, Korea University, Seoul, Republic of Korea; 2 Smart Media Service Research Center, Korea University, Seoul, Republic of Korea; 3 School of Media and Communication, Korea University, Seoul, Republic of Korea

**Keywords:** Psychology, Engineering

## Abstract

This study proposes a novel spatiotemporal crowdsensing and caching (SCAC) framework to address the surging demands of urban wireless network traffic. In the context of rampant urbanization and ubiquitous digitization in cities, effective data traffic management is crucial for maintaining a dynamic urban ecosystem. Leveraging user mobility patterns and content preferences, this study formulates an offloading policy to alleviate congestion across urban areas. Our approach uses an AI-based method at the cell level, providing a practical and scalable solution that can be readily adapted to bustling metropolitan areas. The implementation of our model demonstrated its effectiveness in reflecting real-world urban dynamics, resulting in significant reductions in peak-hour traffic and robust performance across diverse urban settings. The deployment strategy initiates from densely populated transportation hubs, gradually expanding to broader urban areas. This systematic expansion adheres to a policy framework that emphasizes data privacy and sustainable urban development, ensuring alignment with societal needs and regulatory frameworks. By addressing technological efficacy and societal impact, this study enhances the understanding of urban wireless traffic management. It offers mobile network operators, policymakers, and urban planners a comprehensive strategy to harness the potential of spatiotemporal technology, thereby ensuring that cities remain dynamic, efficient, and well-prepared for the future of digital connectivity.

## Introduction

The internet’s widespread availability has fundamentally transformed media consumption, fostering user expectations for instant access to online content^1^. In recent years, mobile content streaming through over-the-top (OTT) platforms has gained significant traction^2^. OTT subscribers now value the flexibility to switch seamlessly between devices, enjoying their favorite content indoors and outdoors on mobile devices. Historically, faster mobile networks and the diversification of OTT platforms have fulfilled these user expectations^1,3^. However, current strategies employed by mobile network operators (MNOs) for access point deployment and network radio resource expansion are anticipated to incur higher infrastructure expenses and bandwidth limitations^2^. Despite the active deployment of 5G networks by MNOs, the anticipated low-latency communication performance remains unrealized^4^.

Typically, OTT content consumption exhibits two distinctive patterns—repetitive requests from a sizable user base and spikes in demand at specific times. For these reasons, users often encounter difficulties in accessing high-quality video content via OTT services, especially in crowded outdoor areas or while moving on public transportation^5^. Additionally, since OTT platforms leverage the MNO-established infrastructure, such as broadband networks, the surge in OTT video streaming directly correlates with increased mobile network traffic. According to the Ericsson report^6^, there was a 36% increase in mobile network data traffic between the first quarter of 2022 and the first quarter of 2023, with video consumption identified as a primary driver behind this substantial increase.

Balancing traffic demand and supply presents a formidable challenge, even in regions with robust mobile network infrastructure^7^. Throughout the day, user traffic fluctuates, leading to pronounced differences between peak and off-peak hours^8^. The primary reason for the traffic imbalance between peak and off-peak hours is the demand for high-quality services^8,9^. However, enhancing the speed and performance of mobile networks poses challenges due to physical and economic constraints^2^. Traditional methods, such as the addition of base stations and the expansion of bandwidth, have become insufficient to keep up with demand due to rising infrastructure costs and limited resources^10^. Consequently, during peak periods, users often encounter difficulties in accessing mobile network services such as OTT due to congestion in cells served by a single base station, resulting from a higher volume of users^11^.

It is serious enough that many users are currently facing challenges in ensuring the quality of service (QoS) for OTT over mobile networks, primarily due to limitations within the existing mobile infrastructures^12^, but the deteriorating performance of mobile networks also poses a hindrance to economic growth and development^13^. For instance, a previous study highlighted that inadequate broadband access could impede the effectiveness and profitability of infrastructure development and investment initiatives, thereby limiting the emergence of new businesses^14^. This underscores the necessity of developing mobile network-related strategies considering both spatial and temporal aspects to effectively mitigate traffic imbalances^11^. Given that user mobility shapes content consumption patterns, which in turn influence user content preference^11,15^, user mobility patterns are the significant factor in establishing content placement strategy^15,16^.

Wireless mobile caching is regarded as an attractive alternative telecommunication technology^7^. Wireless mobile caching can alleviate peak-hour traffic demands by locally storing frequently requested content on users’ devices during off-peak periods and facilitating content sharing between proximate users, thereby significantly reducing duplicate content requests^17,18^. The extent of traffic reduction depends on the storage allocated by users for this purpose^19^. As storage costs continue to decline and smartphones with capacities up to 1 TB emerge, users’ concerns about running out of storage are expected to diminish, enhancing the feasibility of this approach^20^.

In the context of wireless mobile caching, storage and device-to-device (D2D) communication technologies assume pivotal roles^21^. Firstly, the quantity of stored contents directly corresponds to local storage capacity^22^. The ongoing decline in storage cost and rise in mobile device capacity enhance the cost-effectiveness of caching technology^20^. Secondly, D2D enables direct content sharing between closely located user devices without the need for routing through a base station^23^, effectively reducing radio access link loads. Particularly in densely populated areas, D2D links offer significant advantages for peer-to-peer content sharing^21,23^. The frequent utilization of D2D sharing ultimately alleviates the burden on the radio access link, enhancing the accuracy of predicting users’ content consumption patterns^18^.

To better align the stable capabilities of fixed mobile network infrastructure with the dynamic nature of user mobility and content preferences, it is imperative to embrace cooperative caching policies. These policies, which account for both temporal and spatial dimensions, serve to enhance performance^24^. Hence, this study proposes the adoption of spatiotemporal crowdsensing and caching (SCAC) as a desirable cooperative wireless mobile caching policy. SCAC involves transferring frequently accessed content from congested cells via D2D communication, thereby lessening reliance solely on radio access links. Furthermore, SCAC underscores the importance of monitoring user population dynamics across extensive networks.

Previous research on mobile caching highlights the critical importance of achieving a high content-sharing hit-ratio, which is influenced by both user mobility and content preferences^16,18,21,22^. However, pre-storing content to accommodate diverse user preferences encounters challenges due to the complexity of predicting individual behaviors and mobility patterns^25–27^. Since users engage with mobile networks while on the move, user mobility significantly impacts content sharing, highlighting the need to anticipate user mobility patterns^25^. Additionally, successful content placement depends not only on predicting densely populated user congregations or remote user locations but also on considering the frequency of interactions between users^28^. The frequency of user interactions directly influences content-sharing attempts^16^. Therefore, optimizing the performance of wireless mobile caching networks requires strategically placing content to anticipate user movement toward densely populated areas or increased distances. These strategies must consider constraints such as data transmission speed and user distribution^29^.

In wireless mobile caching for mobile OTT services, gathering comprehensive data on user location and content preference is crucial^30^. This extensive data collection significantly enhances the quality of mobile OTT experiences and ensures uninterrupted content delivery, especially during peak hours^31^. Crowdsensing, which leverages the widespread availability of smartphones and devices with built-in sensors, automatically and effectively collects such data^32,33^. Extracting mobility and OTT viewing data from mobile device sensors through crowdsensing enables adaptive media placement and boosts wireless mobile caching network performance, thus reducing reliance on fixed network infrastructure for smoother media consumption^34^. However, while crowdsensing offers significant benefits for data collection and system optimization, it raises security and privacy concerns^35^. Exposing user content preferences and mobility data to unauthorized parties poses a potential risk of personal information infringement. Therefore, crowdsensing strategies in media caching require robust security protocols and transparent privacy policies to mitigate risks.

Additionally, analyzing wireless mobile caching is complex because of the dual challenge of space–time dependency in data traffic, involving high-speed links and massive connections. To ensure efficient network operation across diverse cell environments, a multi-cell strategy is needed that addresses both spatial and temporal dimensions of radio resource management^36^. Given that existing techniques focus on enhancing user interactions and content management within single-cell environments, they fail to leverage user dynamics across multiple cells. This limitation hinders effective congestion management and resource allocation for caching. In this study, we advocate the SCAC principle, which leverages spatial and temporal user information to enhance data collection and caching strategies. This principle is designed to align mobile network infrastructure capabilities with the dynamic nature of user mobility and content preferences. SCAC reduces congestion and potential traffic in cells by facilitating collaborative caching in adjacent cells. Specifically, BSs in less congested cells cache content for users heading toward more congested areas, thereby mitigating traffic spikes. As users transition between cells, this proactive approach promotes a more uniform distribution of content, effectively preventing bottlenecks. Therefore, analyzing local user mobility patterns is essential for this strategy because it provides valuable insights into optimal content placement and its impacts on system-level traffic demands.

**Figure 1:**
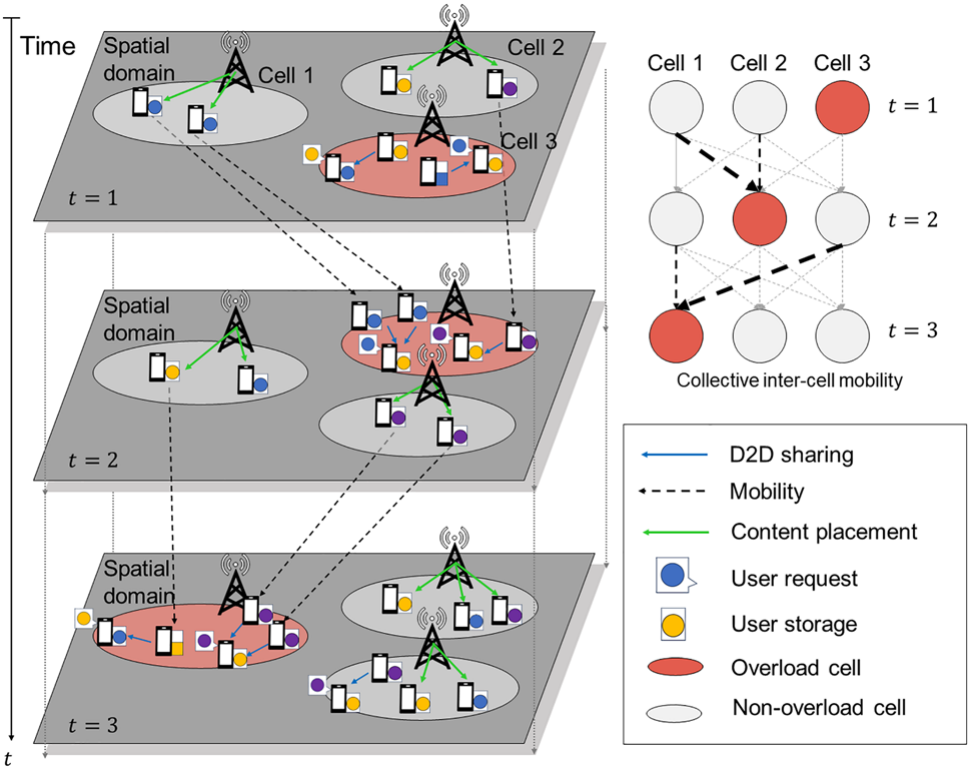
Caching strategy with spatiotemporal information over cellular network configurations.

Figure 1 illustrates the SCAC mechanism across the three cells. Each cell uses crowdsensing to capture unique OTT viewing patterns and user mobility, allowing for predicting future demand during periods of anticipated overload. When a cell predicts an overload, it preemptively caches content likely to be needed by incoming users, thereby directly addressing anticipated demand through targeted content delivery. This proactive approach facilitates a spatiotemporal distribution of traffic, efficiently preventing cell overloads without additional infrastructure. However, as the system scales up, content placement becomes more complex because of the increased computational burden on the centralized network coordinator. This challenge is compounded by the inefficiency of tracking individual user mobility and preference trajectories on a large scale. In addition, continuous real-time monitoring of users raises privacy concerns because it may disclose sensitive personal details, such as residence information^35^.

To address privacy concerns, user trajectories can be managed as anonymized cell-level transition logs instead of detailed individual tracking. This approach captures inter-cell user transitions without the need for individual user identification within each cell, thereby effectively safeguarding user data privacy^26^. The aggregated approach significantly influences how base stations interpret movement patterns. While individual users exhibit diverse schedules and behaviors, the base station that observes the collective population only perceives inter-cell transitions, effectively treating the user group as a whole. This perspective reduces the significance of individual patterns, emphasizing characteristics solely dependent on the current state of collective movement. In this context, the resultant user mobility is modeled as a cell-level transition model. The collective inter-cell population flow is predicted and exchanged across the multi-cell network. Establishing a collaborative approach through inter-cell cooperation is appealing for practical deployment and reliable operation.

Based on the discussion above, this study aims to empirically evaluate the efficacy of SCAC in addressing mobile traffic issues using a simulation platform that captures real-world mobility patterns, preferences, and population dynamics. By employing simulations enriched with real-world mobilities and preference data, this study tries to provide empirical evidence supporting the efficacy of SCAC in alleviating cell-request overloads and related costs. Such findings will provide tangible benefits for both MNOs and users. Thus, this study proposed the following research questions:

- RQ1. Is SCAC a reliable solution for addressing mobile traffic issues?
- RQ2. Under what conditions or environments can effective implementation of SCAC be achieved?

## Results

The effectiveness of SCAC in reducing mobile network traffic and its impact on the network environment were assessed in a simulation in an urban area with a user population of 400,000.

### Assessing SCAC’s reliability in addressing traffic issues (RQ1)

**Figure 2:**
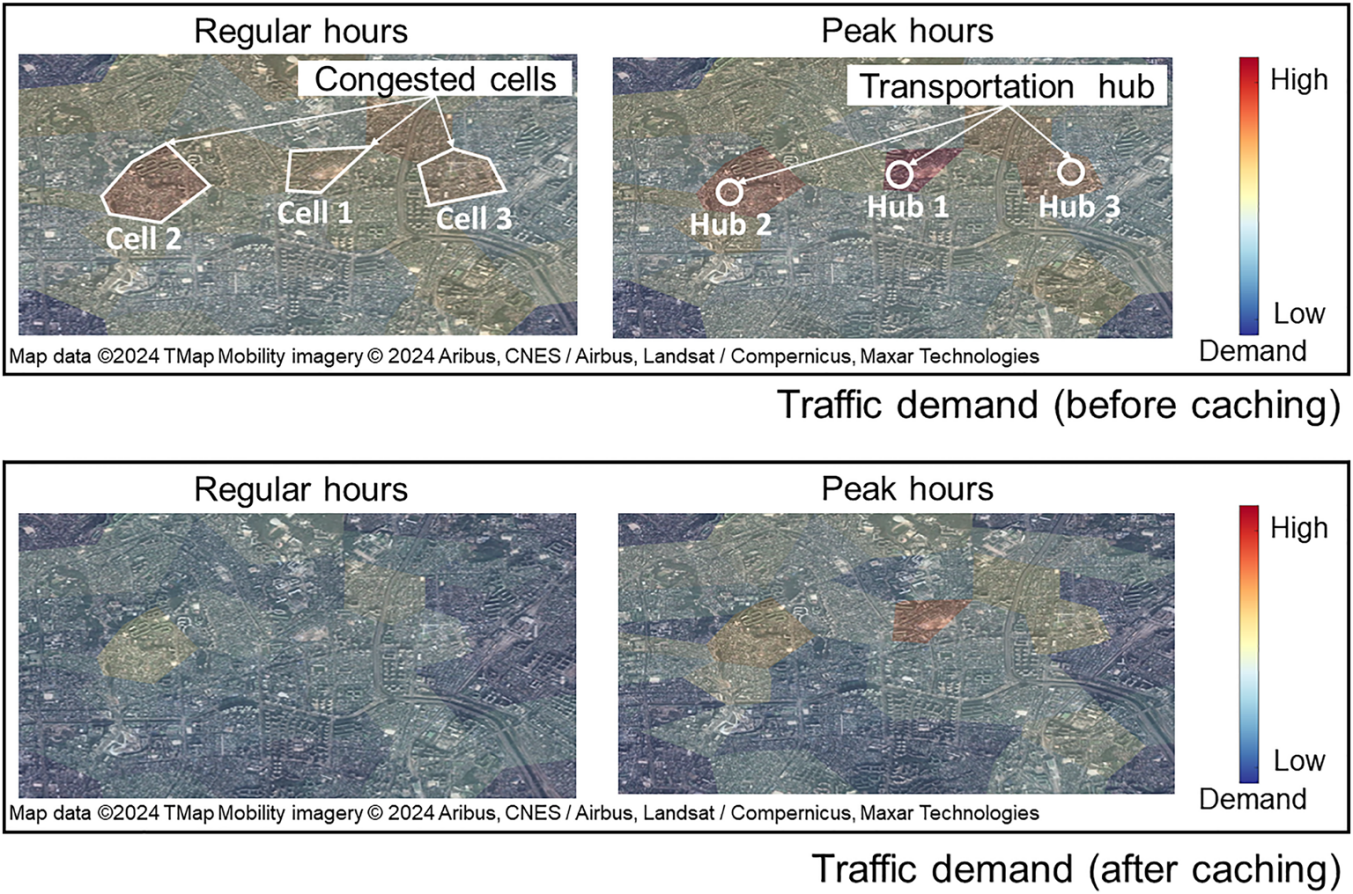
Spatiotemporal offloading results during the regular and peak hours.

Figure 2 shows the effectiveness of the SCAC strategy in terms of offloading gain: traffic demand was relieved after content sharing. Data traffic and user populations are normalized to the highest value in Cell 1, while the offloading ratio is expressed as the percentage of demand alleviated through sharing among users. Color coding in each cell represents the amount of traffic demand before and after the caching operation. The color bar shows the traffic demand volume, with red shades indicating high demand and blue shades indicating low demand. The coloring standard for each region is based on the highest value of peak-hour traffic demand. The three cells with the highest average daily demands are highlighted with white borders to illustrate congestion hotspots. Cell 1 experienced peak traffic between 12:00 and 14:00, while other periods were considered to be regular hours. The SCAC strategy demonstrably reduced traffic during both periods. During regular hours, the strategy led to significant reductions in load on cells, evidenced by many blue-demand areas indicating lower traffic. While the offloading gain was slightly lower during peak hours due to high user demand exceeding caching capabilities, the amount of traffic borne by the corresponding cell was still reduced. Furthermore, all congested cells shared a common characteristic: they are located near transportation hubs, which significantly contributes to the higher traffic in these locations.

**Figure 3:**
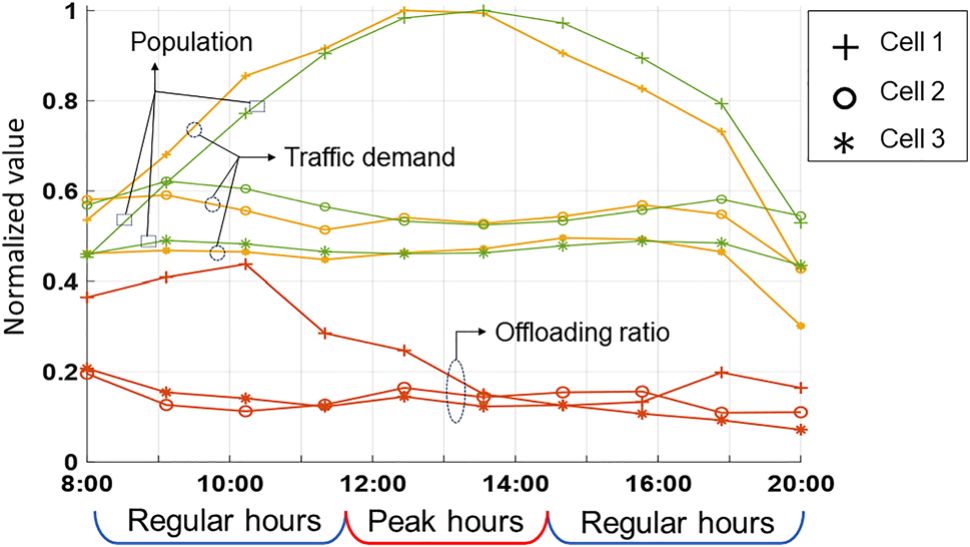
Spatiotemporal offloading results for three congested cells over time-varying daily traffic dynamics.

Figure 3 shows the performance of the cell-level caching strategy, specifically focusing on the offloading gain in three congested cells with the highest average daily traffic demand. It presents data traffic, user population, and offloading gain for each cell at hourly intervals during the day. Data traffic and user populations are normalized to the highest value in cell 1, and the offloading gain is expressed as the proportion of demand alleviated by sharing among users. The distributed offloading strategy at the three congested cells reduces traffic demands by 31.0% during regular hours and 26.4% during peak hours with respect to the original traffic demands. It is observed that traffic demands exhibit patterns similar to hourly population transitions whereas daily patterns of offloading gains are distinct.

Cell 1 experiences a gradual rise in traffic requests in the morning, with peak hour data traffic exceeding double that of regular hours. The SCAC framework anticipates this surge and proactively caches the content expected to be requested during peak hours. This allows users who will be in cell 1 during peak hours to have the content stored even before the peak period, facilitating efficient sharing with the visiting population. This proactive approach enables the early implementation of offloading strategies, effectively securing necessary resources before the peak hour surge. In contrast, cells 2 and 3 experience consistent traffic patterns, resulting in sustained offloading performance due to ongoing collaboration with adjacent cells. As data traffic patterns fluctuate in tandem with hourly population changes, the ability to predict future congested cells both spatially and temporally is facilitated by specifically tracking inter-cell mobility through crowdsensing. This predictive approach to content caching ensures that mobile users heading to high-demand areas can seamlessly share their data with neighboring users, either before reaching their destination or immediately upon arrival.

### Optimal conditions or environments for effective implementation of SCAC (RQ2)

**Figure 4:**
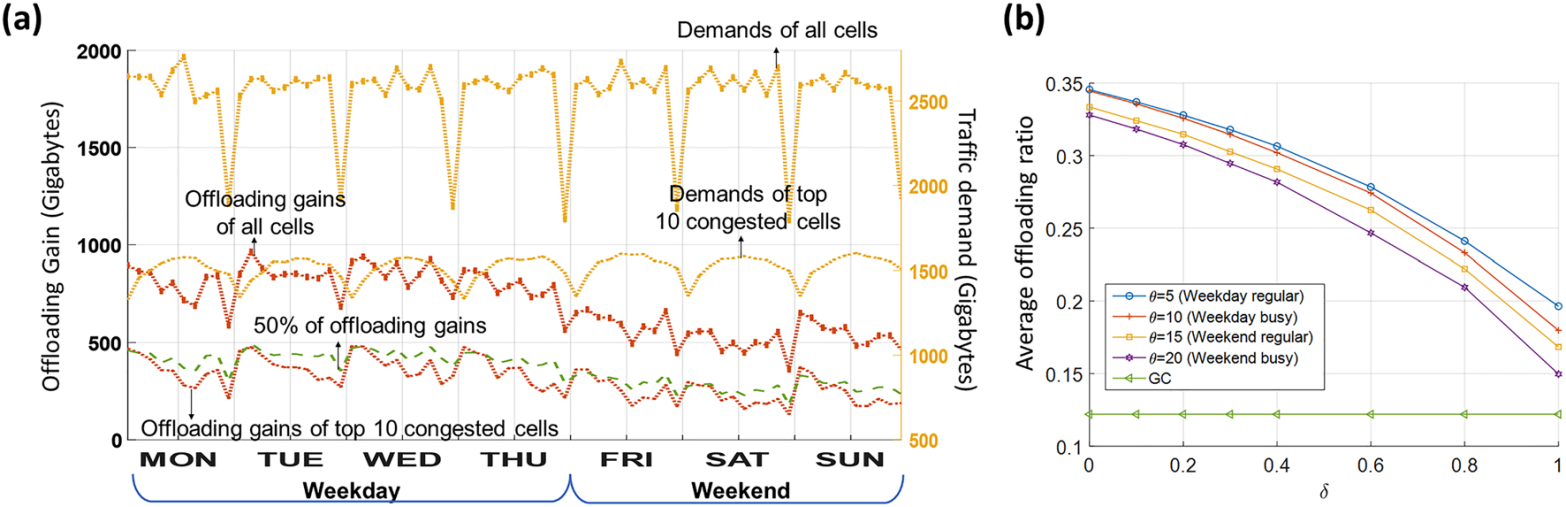
(**a**) Impacts of spatiotemporal caching: transitions of weekly patterns for cell traffic and preference distributions via spatiotemporal consideration over urban real-map configurations. (**b**) Average offloading gain performance with inaccurate mobility information.

Figure 4(a) presents a comparison of the weekly variations in gross content consumption and offloading gain, evaluated in gigabytes (GB). Monday through Thursday are classified as weekdays, and Friday through Sunday as weekends, reflecting user behavior patterns. On Fridays, people often leave work early and prepare for the weekend, resulting in demand patterns that differ from typical weekdays. Demand volume (yellow line) is measured on the right y-axis, and offloading gain is measured on the left y-axis. The total demand volume remains steady throughout the day with regular drops late at night, while the offloading gain exhibits minimal daily variation. The volume of content shared with users through content placement amounts to 818.27 GB on weekdays and 543.26 GB on weekends per hourly regular operation interval. Regarding content preference patterns, the local preferences observed on weekdays closely mirror the global preferences. This observation suggests that during weekdays, individual content preferences tend to synchronize as users move and interact socially^37^. To reflect this difference in preference patterns, the content propagation degree $\theta$ is set to 5–10 on weekdays and 10–20 on weekends for simulation under various environments. The strategic placement of popular content results in enhanced sharing performance on weekdays compared with weekends. Although the offloading gain decreases slightly when $\theta$ is higher on weekends, it remains effective. This is because more diverse content is stored to support a wider range of preferences during weekends.

This framework ensures consistent offloading gains by adapting to variations in traffic and user preferences. The uneven regional distribution of traffic demands causes congestion to concentrate in no more than 10 cells. To balance data traffic, the trained SCAC principle cooperatively attempts to spread traffic demands over adjacent cells. Thus, the sum of the offloading gain of these cells covers half of the total offloading gain. The offloading gain indirectly shows bandwidth savings, as it reduces the load on network infrastructures and improves QoS by utilizing saved resources. Additionally, localized caching reduces the distance data must travel, thereby improving latency and enhancing overall network efficiency.

Figure 4(b) depicts the impact of the availability of user mobility information under various data traffic configurations. Real-world systems typically lack complete user mobility due to the inherent difficulty of continuously pinpointing the user’s instantaneous location. The completeness of the collection about user mobility can be categorized as known or unknown. To characterize this, the incompleteness parameter, denoted by $\delta$, is introduced as the percentage of users with unknown mobility patterns. The destinations of users of unknown mobility are chosen uniformly toward adjacent cells. For the users comprising the $\delta$ proportion with unknown mobility, their destinations are chosen uniformly among adjacent cells. Two categories of data traffic configurations were assessed: regular versus peak hours and weekdays versus weekends. Each is characterized by four different values of the content propagation degree $\theta$= 5, 10, 15, and 20. With complete user mobility information $(\delta =0)$, sharing gains are maximized. With increasing $\delta$ (incompleteness parameter), the proportion of content volumes determined by random placement increases, resulting in incomplete content sharing and a lower hit ratio in the destination cells of user mobility. For comparison, the sharing results outperform the geographic caching solution, which only considers the state within a cell without taking into account spatiotemporal mobility information. This demonstrates that even the utilization of inaccurate spatiotemporal information is effective in enhancing content accessibility.

## Discussion

In urban regions, user mobility varies between densely and sparsely populated areas, often linked to transportation hubs, leading to imbalances in mobile network traffic^9^. Addressing these imbalances requires a flexible caching strategy that considers both spatial and temporal factors. This study aimed to achieve two objectives: first, to assess the effectiveness of the SCAC framework in decreasing bandwidth demands, particularly within congested cells, through collaboration with neighboring cells; and second, to investigate the commercial viability and explore the policy support mechanisms required to expedite the adoption and scaling of the SCAC principle in transportation hub areas.

Transitioning from a controlled simulation environment to real-world applications requires considering scalability in diverse and larger urban environments. Real urban environments can introduce new preferences and mobility patterns not previously encountered. For instance, sports stadiums may exhibit patterns where content related to the game is consumed intensively. Therefore, it is advisable to implement SCAC in pilot projects focusing on specific cohorts (e.g., university campuses, sports stadiums, and densely populated transportation hubs) to evaluate the potential of these strategies in alleviating data traffic volumes and ensuring a smooth transition to larger scales.

Considering the minimal base station transitions observed at the macrocell level^8^, it may be reasonable to confine the cooperative cells in the pilot to adjacent cells. Restricting crowdsensing activities to pre-peak hours could optimize traffic distribution during congested periods. Performance benchmarks demonstrate that the pilot operates effectively with only 30% of mobility data, eliminating the need to capture all user patterns. Evaluating the pilot project in transportation hubs will reveal the potential for traffic reduction with complete mobility information. Insights garnered from this evaluation will guide the expansion to other high-traffic areas, validate the technology’s scalability, and establish infrastructure guidelines for collaborative cells. This approach facilitates a cascading expansion to effectively support broader urban zones.

Cell-level user mobility is tracked through handover technologies, which monitor population movements within a cell and to adjacent cells. In South Korea, for example, KT collects and shares mobility data based on LTE and 5G signals at the district and traffic polygon levels in hourly intervals^38^. This means that SCAC can sufficiently track cell-level user mobility using existing infrastructure during the operational interval. While individual tracking might encounter issues like the ping-pong effect, SCAC estimates mobility at the base station level, mitigating short-term inaccuracies. Implementing SCAC requires initial investments in data collection, processing, and operating trained neural networks, with long-term costs such as maintenance and data storage analyzed post-pilot. However, SCAC’s spatial and temporal distribution of network resources is expected to reduce the need for additional infrastructure to meet growing demand, offering potential cost-saving benefits.

Traditionally, MNOs have addressed the surge in mobile traffic by enhancing macrocells through the incorporation of additional cells, antennas, and network automation. However, with the available spectrum in commonly used frequency bands nearing full utilization, a constraint exists on the capacity expansion of macrocells. Consequently, a shift toward higher frequency bands such as the 26 GHz has become necessary. While this higher frequency band holds promise for increased capacity, it exhibits limited propagation distance and is better suited for compact, street-level small cells rather than large macro cells^9,39^. The transition from macrocells to small cells in mobile networks has become essential because of the limitations posed by full spectrum utilization in commonly used frequency bands. Smaller cells, which inherently cover smaller geographical areas, exhibit lower user density than macrocells. Consequently, the adoption of small cells reduces the crowdsensing burden per cell. Incorporating SCAC’s content placement strategy into small cell infrastructure improves operational efficiency and addresses the critical need for sustainable and adaptable traffic management in densely populated urban areas.

Furthermore, SCAC’s adaptability extends to incorporating mobile edge computing (MEC). MEC brings computation and storage resources closer to the user, reducing latency and improving response times^40,41^. By integrating MEC with SCAC, we can further enhance content caching and delivery, ensuring the framework remains effective even as network architectures evolve. Additionally, the introduction of local storage-equipped helpers can enhance SCAC's local sharing performance^42^. These helpers, which may include fixed devices or mobile entities like vehicles, improve the overall efficiency of content distribution. This evolution in network management illustrates a significant shift toward more user-centric and demand-responsive telecommunication policies. By prioritizing efficient data distribution and network utilization, SCAC underscores the potential for a more balanced digital ecosystem. Given the ongoing complexities of urban data traffic and user demands, the integration of such forward-thinking strategies is imperative for fostering a more equitable and efficient digital landscape.

The efficacy of SCAC can be further improved by incorporating more detailed user data, including content preferences and viewing history, into the user mobility information. However, the collection and handling of such personal information can lead to sensitive concerns. Users are growing more cautious about granting consent for location data collection, especially considering the COVID-19 pandemic^43^. This study, which conducted a systematic literature review of data privacy issues with contact tracing applications, highlighted significant privacy concerns regarding the collection of personal data, including location information. It emphasizes the need for clear privacy policies and transparency to address public concerns. This heightened sensitivity arises from concerns related to tracking the locations of infected individuals^43,44^. The risk of MNOs collecting an excessive volume of mobility data emphasizes the importance of institutional measures. Stringent legislation should be enacted to clearly define the permissible boundaries of data collection. These measures, enforced with penalties for violations, should extend beyond mobility data to encompass other personal datasets, such as OTT viewing metrics. While MNOs currently have access to mobility data, OTT platforms possess unique datasets related to viewing habits, albeit not necessarily location information. The convergence of these two types of information, which are crucial for maximizing the effectiveness of SCAC, indicates that collaborative efforts between MNOs and OTT platforms could be pivotal in genuinely commercializing SCAC.

Ongoing global disputes between MNOs and OTT platforms revolve around network usage fees related to the traffic generated by OTT content streaming. These disputes highlight persistent concerns regarding network neutrality and the equitable distribution of network maintenance costs^45^. Moreover, South Korea’s representative MNOs failed to meet the performance conditions for the allocation of 5G frequency spectrum. These conditions included deploying a sufficient number of cell sites for 5G services and investing adequately in delivering 5G services over the 28 GHz band, which were commitments made by the MNOs at the inception of their operations. This failure led to the cancelation of their 28 GHz licenses, impeding the fulfillment of user demands for mobile network services, including those of the OTT service. Given these difficulties, implementing SCAC presents a mutually beneficial solution for MNOs and OTT platforms, alleviating financial burdens through reduced infrastructure and data transmission costs. In addition, the adoption of SCAC opens up possibilities for innovative partnerships. Through collaborations, SCAC collaborative efforts leading to improvements in OTT quality and the introduction of new mobile data plans not only expand the user base and improve market appeal but also strengthen the presence of both MNOs and OTT platforms.

However, the reliance of SCAC on pre-allocated user storage inherently requires active user engagement. This approach may encounter resistance from users who are cautious about sharing device storage or personal data. Research on users’ psychological barriers to mobile caching suggests that potential user resistance may not be as great as feared. Especially when the benefits of participation, such as an improved content viewing experience, are effectively communicated to users^46^. Specifically, the simulation settings of the SCAC framework, which limit data usage to 3 GB, are reasonable and reflect realistic usage scenarios that are palatable to users. Additionally, providing appealing incentives as part of a user-centric approach can further mitigate these concerns and encourage participation^47,48^. Incentive strategies can facilitate the establishment of a harmonious relationship among MNOs, OTT platforms, and users, thus contributing to the creation of a mutually beneficial digital landscape for all parties involved.

Moreover, measuring user satisfaction with the SCAC framework would ideally be conducted post-deployment. Preliminary research indicates that user acceptance tends to increase when they are aware of the limitations of existing infrastructure and the potential improvements offered by new technologies like mobile caching^49^. Given the rising number of video streaming service users and the growing data demand, the user-centric approach of the SCAC framework, utilizing user preferences and mobility data, is likely to be well-received.

The limitations of the research and suggestions for future work are also presented. Implementing the SCAC principle involves forecasting collective statistics related to user mobility and content preferences. Dense public transportation networks characterized by regular schedules and established routes alleviate the need for precise predictions of collective user dynamics and temporal variations. However, tailoring SCAC strategies to align with the distinctive dynamics of user populations using public transportation can yield significant caching benefits. Consequently, further research examining the dynamics of public transportation mobility and analyzing content consumption patterns during public transportation usage is essential to enhance the efficacy of SCAC strategies.

Although SCAC strategies demonstrate potential in enhancing overall performance at the cell level, a more nuanced investigation is required to validate how content is shared among individual users and to ensure equitable content distribution among all users. The unpredictable nature of user behaviors and fluctuating network conditions pose challenges in achieving fair and efficient content distribution. Addressing these challenges necessitates the development of a robust content-sharing protocol that accommodates this variability. This protocol must ensure reliable and efficient content sharing, even under non-ideal conditions, such as weak device connections or sporadic user cooperation. Adapting the protocol to consider the actual availability of content sharing under diverse real-world conditions significantly improves the efficiency of content distribution among users.

Space–time considerations offer new avenues for collaboration between MNOs and OTT platforms in developing cooperative management strategies that optimize network resources without substantial infrastructure investments. Consequently, the development and implementation of innovative business strategies in this realm are crucial areas for further investigation to ensure the economic viability and market acceptance of these technological advancements. However, for these possibilities to materialize into actual business ventures, empirical exploration is essential to determine the optimal conditions under which two service providers can form a strategic alliance and understand the resulting consequences.

## Methods

This section details the design of a testbed that leverages datasets comprising user mobility and content preferences. An AI-based framework that employs neural parameter optimization to enhance content delivery efficiency is proposed. Specifically designed to optimize the selection of content stored on the user’s device, this framework enhances the efficiency and accuracy of content delivery at the cell level. This integrated approach leverages advanced data analysis and adaptive caching strategies to enhance the user mobile OTT experience and network performance.

### Urban virtual testbed environment

**Figure 5:**
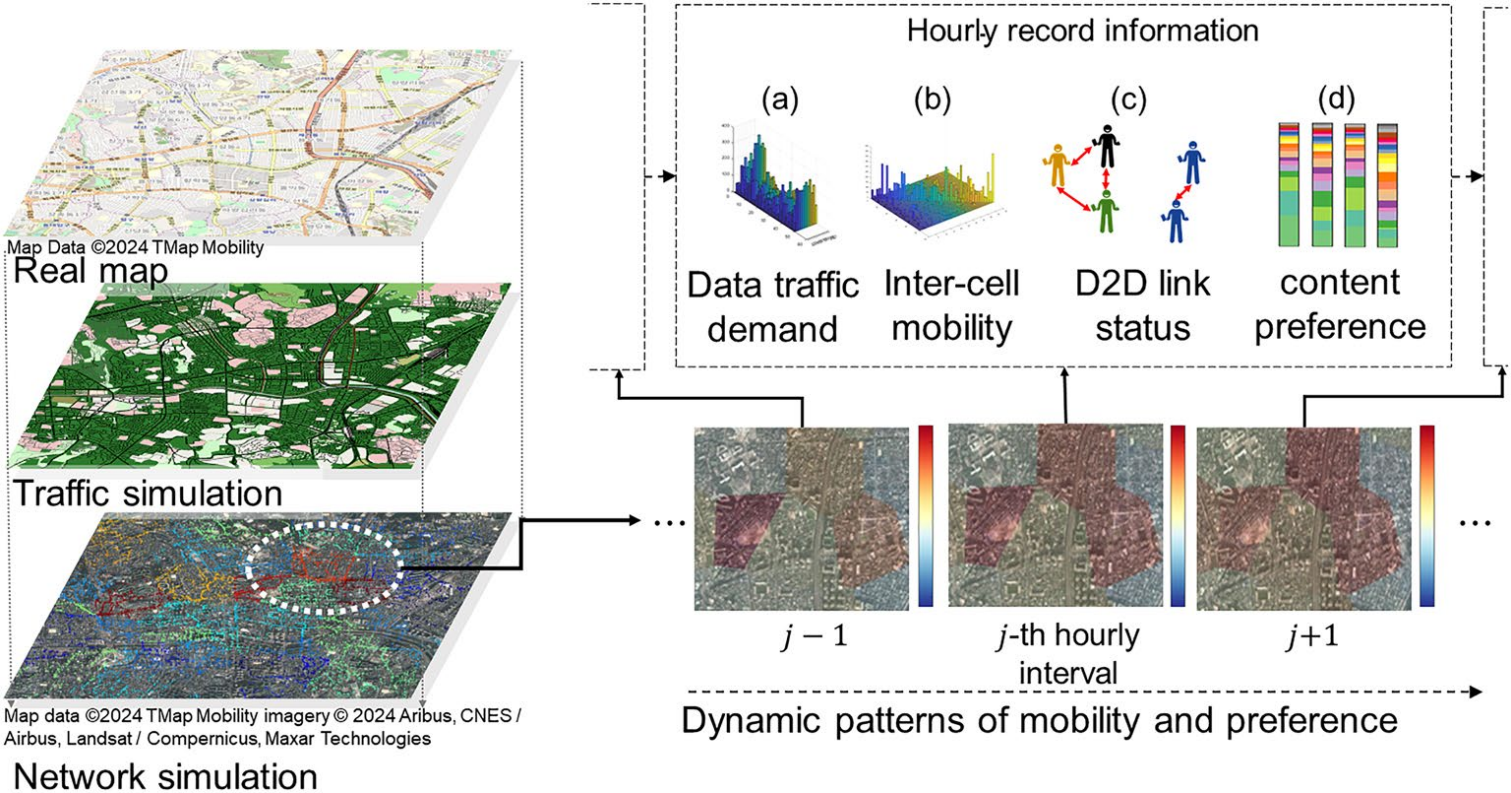
Preference and mobility data collection process in an urban virtual testbed.

The testbed leverages a dataset comprising user mobility and content preference to capture transition behaviors and collective movement patterns. Figure 5 shows the data collection process. User mobility was tracked using an open-source traffic simulator, simulation of urban mobility (SUMO^50^), within a 5 × 5 km urban area near the Korea University campus in Seoul. Fifty real macrocells were selected based on geographical information, and a population of pedestrians and vehicles was distributed proportionally to the actual demography. Daily dynamic patterns are tracked from 8 a.m. to 8 p.m., reflecting that on-the-go user content consumption is concentrated during the daytime. Individual trips along the streets are headed to destinations chosen according to mobility patterns. To accurately depict mass mobility patterns in large-scale simulations, it is essential to begin with the meticulous modeling of individual mobility patterns, drawn from realistic data. The simulation monitors the instantaneous measurement of data traffic demand, inter-cell mobility, D2D link status, and content preference.

### Preference and mobility collection

User content preferences serve as authentic reflections of user behavior. To enhance the accuracy of our analysis, we adopt preference models from previous studies. Leveraging a content library comprising the top 100 ranked content, we categorize users into two groups: regular and heavy content consumers. The viewing patterns for content depend on temporal preference patterns, which exhibit regional variations. Previous studies have explored the use of a shuffled content library to represent geographically heterogeneous preferences^51^. Building on this concept, we developed a model that gradually propagates regional preference patterns, initiating from densely populated areas. Geographical propagation of preferences can be characterized in a one-dimensional manner by introducing the propagation degree, denoted as $\theta$. This degree represents the norm of the difference in popularity rank vectors. For example, an increase in $\theta$ value signifies more pronounced regional preference characteristics, highlighting clearer distinctions in content preferences across different regions. This variability emphasizes the strategic importance of content placement from a network perspective. At the cellular-level, user mobility is tracked through inter-cell handover technology, which monitors both the population within their range and those transitioning to neighboring cells^52^. The regular collection of mobility and local preferences occurs at the closest cell, providing insights into level user behavior. Meanwhile, global preferences are aggregated across the network to identify broader patterns.

To enhance content placement intelligence, inter-contact model parameters^28^, such as average user dwell time and average user interaction time, are explicitly calculated and incorporated as input data. Hourly traffic demands represent the total volume of content requested within each cell per hour. The supply of each cell is set as the average of its demands over the entire period. Furthermore, link configurations for content sharing are subject to the wireless characteristics of urban-model channel propagation, with link status modeled by the probability of users coexisting in a cell^16,28^. For our simulation, D2D communication is generally restricted to devices with 3 GB storage within a 20 m distance. This general setup reflects typical interactions within the wireless caching network, offering a standardized environment for analyzing network dynamics and content sharing. The simulation mobility patterns mirror real-world dynamics: (i) limited mobility: over half of the population remains within a single cell. This result confirms a previous study on mobility^8^. (ii) Hub concentration: users congregate at transportation hubs reflecting high-traffic areas.

### Neural parameter optimization

A distributed solution for content placement inherently involves optimization for the dynamic nature of user mobility and content preference. Various network optimization techniques have achieved success, including a recent AI-based approach that effectively leverages temporal patterns revealed from historical input records^53,54^. In this subsection, we explore a neural network model that processes spatiotemporal characteristics of urban environment datasets.

**Figure 6:**
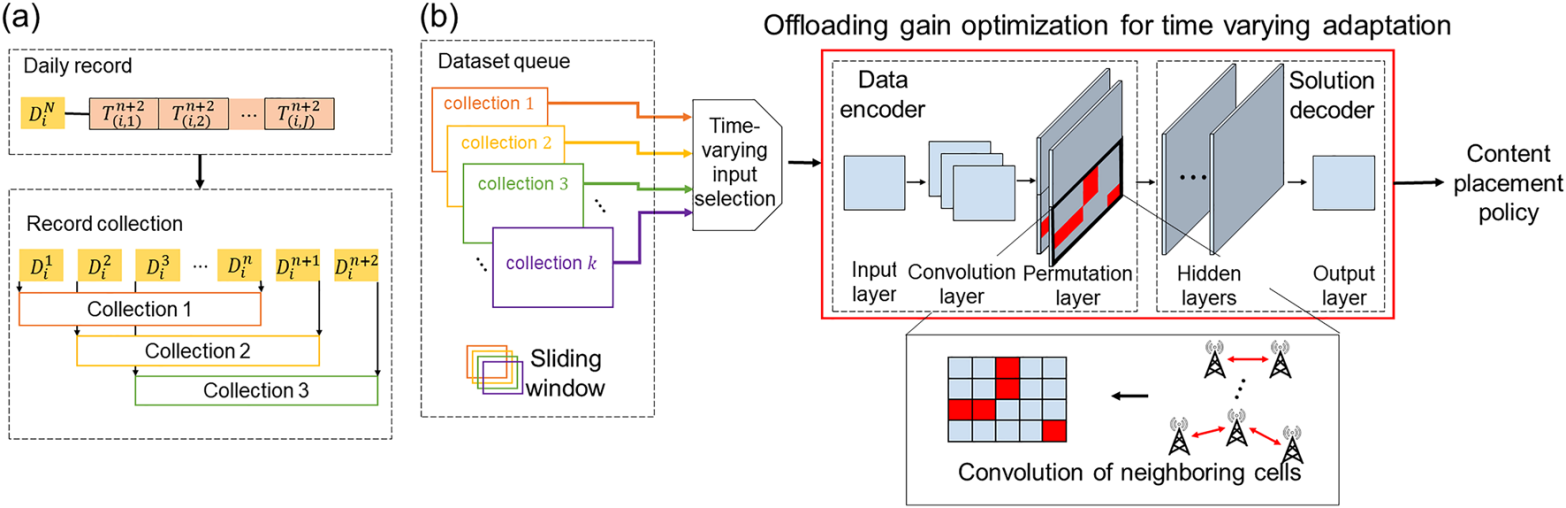
(**a**) The data preprocessing process. (**b**) Neural network model.

Figure 6(a) illustrates the process of aggregating hourly records from the mobility and preference domains into daily records, which are subsequently stored as data collections in a dataset queue. The record that cell $i$ processes at the $j$-th hourly interval of the $n$-th day is denoted by ${T}^{n}\left(i,j\right)$. The resulting daily records ${D}_{i}^{n}$ encapsulate time-varying measurements related to data traffic, content preference, and mobility patterns within the cell-level operating cycle. Over extended periods, such as 10 days, these daily records, which are stored as data collections in a dataset queue, serve as input batches for training the content placement strategy.

Figure 6(b) presents the structure of a neural network model that constructs computation rules for spatiotemporal offloading without requiring exact knowledge of global network states. The model comprises two major components: a data encoder and a solution decoder. The data encoder includes an input layer, a convolutional layer, and a permutation layer, whereas the solution decoder contains several layers of dense neural networks and an output layer. Data collections stored in the dataset queue are fed into the input layer as training datasets. These collections enable training for time-adaptive operations that adapt to weekly and monthly user behavior patterns. Batch normalization was applied to avoid issues caused by significant regional differences in data traffic and population density. The convolutional layer extracts the space–time characteristics of the input records. The extracted geometric feature constructs a latent space for location-specific traffic and preference records. The permutation layer associates the extracted local patterns with the adjacency information of the real-map geography and encodes traffic and preference flows. Given that user population and data traffic exchanges are infrequent between distant cells, these geographical features can be encoded as an adjacency map. This map is masked to the convolution layer output to calculate additional convolution only among cells with user and traffic exchanges. In addition, a permuted tensor is applied to provide equivariance for input ordering. In contrast to image processing, where pixel order matters, this feature ensures that the optimization solution remains independent of the location information of cells. Consequently, the developed model encodes network dynamics features for cell-specific information independent of cell-specific geography. The solution decoder comprises several layers of dense neural networks and an output layer. In forward-pass computations, the decoder network calculates the offloading gain of individual cells. It is defined as the total sum of the remaining demand after offloading. Furthermore, backpropagation computations update parameter sets via the RMSProp optimizer^55^. The output layer yields a list of content volumes placed on the user population residing in the cell.

## Data Availability

Sample files of SCAC mobility data were uploaded in the repository. They can be found at: https://github.com/happywater12/SCAC
